# Effective Non-Radiative Interfacial Recombination Suppression Scenario Using Air Annealing for Antimony Triselenide Thin-Film Solar Cells

**DOI:** 10.3390/ma17133222

**Published:** 2024-07-01

**Authors:** Rong Tang, Wenyong Hu, Changji Hu, Chunyan Duan, Juguang Hu, Guangxing Liang

**Affiliations:** 1 School of New Energy and Environmental Protection Engineering, Foshan Polytechnic, Foshan 528137, China; 2 Shenzhen Key Laboratory of Advanced Thin Films and Applications, Key Laboratory of Optoelectronic Devices and Systems of Ministry of Education and Guangdong Province, State Key Laboratory of Radio Frequency Heterogeneous Integration, College of Physics and Optoelectronic Engineering, Shenzhen University, Shenzhen 518060, China

**Keywords:** antimony triselenide, air annealing, thin-film solar cells

## Abstract

Antimony triselenide (Sb_2_Se_3_) has become a very promising candidate for next-generation thin-film solar cells due to the merits of their low-cost, low-toxic and excellent optoelectronic properties. Despite Sb_2_Se_3_ thin-film photovoltaic technology having undergone rapid development over the past few years, insufficient doping concentration and severe recombination have been the most challenging limitations hindering further breakthroughs for the Sb_2_Se_3_ solar cells. Post-annealing treatment of the Sb_2_Se_3_/CdS heterojunction was demonstrated to be very helpful in improving the device performance previously. In this work, post-annealing treatments were applied to the Sb_2_Se_3_/CdS heterojunction under a vacuum and in the air, respectively. It was found that compared to the vacuum annealing scenario, the air-annealed device presented notable enhancements in open-circuit voltage. Ultimately a competitive power conversion efficiency of 7.62% was achieved for the champion device via air annealing. Key photovoltaic parameters of the Sb_2_Se_3_ solar cells were measured and the effects of post-annealing treatments using different scenarios on the devices were discussed.

## 1. Introduction

Solar energy, which is considered as one of the most important renewable energy sources, has accounted for about 4% of total global electricity today. It is expected that the contribution percentage will gradually increase to 25% by 2050 [1]. According to the literature [2], silicon-based technology still dominates the PV market by holding approximately 90% of the market share. However, the energy consumption of the silicon wafer fabrication is massive since the applied temperature in the smelting process can go beyond 1400 °C [3]. On the other hand, thin-film technologies have seen significant progress in the last decade as the power conversion efficiency (PCE) of state-of-the-art devices is already comparable to that of silicon-based solar cells. Especially, CdTe and perovskite solar cells have achieved highly competitive PCEs of 22.4% and 26.1%, respectively [4]. Moreover, the GaAs solar cell exhibited an extremely high PCE of 29.1% [5]. However, various degrees of toxicity and the unsatisfactory lifetime of these state-of-the-art thin-film solar cells might be problematic for their commercialization. For instance, elements such as Cd, Pb and As contained in the solar cells mentioned above are considered to be highly toxic to public health [6]. Poor stability is one the most tricky issues hindering large-scale application of perovskite solar cells, although both organic and inorganic halide perovskite solar cells have exhibited remarkable progress in recent years [7,8,9,10]. Among the emerging materials for thin-film photovoltaic technologies, antimony triselenide (Sb_2_Se_3_) is regarded as a promising candidate thanks to its excellent optoelectronic properties. The chalcogenide compound is a low-toxicity material which possesses an optimal bandgap (~1.1–1.3 eV, close to the Shockey–Queisser value), a high absorption coefficient (>10^5^ cm^−1^) and proper carrier mobility (~10 cm^2^ V^−1^ s^−1^). As a one-dimensional crystal–structural material, Sb_2_Se_3_ exhibits highly anisotropic carrier transport in various orientations. It has been widely acknowledged that crystal orientation is one of the most important features for Sb_2_Se_3_ thin-film solar cells. Decent photogenerated carrier transport can only be achieved within the covalently bonded (Sb_4_Se_6_)_n_ ribbons, whereas the transport would become much harder in between the (Sb_4_Se_6_)_n_ ribbons held by van der Waals forces [11]. Over the last few years, effort was put into various aspects (e.g., crystal grain orientation [12,13,14,15,16,17,18,19,20,21], effective doping [16,17], back contact modification [18,19,20] and heterojunction interface engineering [21,22,23]) to boost solar cell efficiency. To date, the highest PCE of the thin-film Sb_2_Se_3_ solar cell has reached 10.57% [24], where the Sb_2_Se_3_ light absorber was prepared using an additive-assisted chemical bath deposition. Still, the efficiency is far lower than the corresponding Shockey–Queisser limit, which is supposed to be over 30% [25]. The champion device reported by Zhao exhibited an outstanding short-circuit current (*J_SC_*) and FF of 33.52 mA/cm^2^ and 67.64%, respectively [24]. Yet there is a notable gap between the open-circuit voltage (*V_OC_*) of 0.467 V and its corresponding Shockey–Queisser value, resulting in a nearly 0.5 V *V_OC_* deficit for the device. In fact, the *V_OC_* deficit has become the most challenging issue not only for the newly emerging Sb_2_Se_3_-based solar cells but also for other more developed thin-film photovoltaic technologies such as CIGSSe, CdTe and CZTSSe [4]. The origins of the *V_OC_* deficit for Sb_2_Se_3_-based solar cells have been significantly investigated and now can be attributed to two main reasons. Firstly, the free carrier concentration in the pristine Sb_2_Se_3_ bulk is naturally low, which ends up with a limited built-in voltage *V_bi_* and thus a low *V_OC_*. Fortunately, the issue can well be addressed once effective p-type doping strategies are applied [16,17]. Secondly, deep defects, especially those at the heterojunction interface of the device, act as recombination centers and lead to severe *V_OC_* loss. Post-annealing treatment is an effective and straightforward procedure that is usually used to optimize the heterojunction interface for chalcogenide thin-film solar cells [17,26,27]. An appropriate post-annealing treatment would promote elemental interdiffusion between the light absorber layer and the buffer layer, thus moderating band alignment and passivating interfacial defects at the heterojunction. For substrate Sb_2_Se_3_ thin-film solar cells, the post-annealing treatment is essential to obtain decent efficiency. Distinct improvements in almost every aspect of the device performance could be observed without and with the annealing process. Such post-annealing treatments were not often reported in those Sb_2_Se_3_ devices with superstrate configuration. However, we believe the mechanisms behind them are quite similar, since the substrate temperatures in the superstrate devices were usually around 300 °C [11,13,15,21], which is close enough to the annealing temperature that we used in our previous works [17]. Therefore, it is very likely that Sb_2_Se_3_ film growth and elemental interdiffusion across the Sb_2_Se_3_/CdS interface occurred simultaneously for these superstrate devices. Generally, the post-annealing treatments were conducted under inert atmosphere (argon or nitrogen) to prevent oxidation of the device [26,27]. Our previous work reported a rapid thermal treatment of the Sb_2_Se_3_/CdS substrate in vacuum condition to mitigate nonradiative recombination near the heterojunction region [17]. On the other hand, post-annealing treatments were also carried out under ambient air for other chalcogenide-based thin-film solar cells such as CIGS and CZTSSe [28,29]. It is believed that the interaction between oxygen and the defect states could increase the effective doping density and passivate harmful deep defects for the devices. Liu stated that the right dosage of oxygen during the Sb_2_Se_3_ thin-film deposition process is beneficial to the device performance [30]. Jakomin applied a moderate air annealing at 175 °C to the NaF/Sb_2_Se_3_ interface to promote sodium diffusion to the Sb_2_Se_3_ bulk film. As a result, the *V_OC_* of the solar cell was increased due to suppressed recombination within the device [31]. However, post-annealing in ambient air for the Sb_2_Se_3_/CdS heterojunction has been rarely reported. In this work, post-annealing treatments were applied to the Sb_2_Se_3_/CdS heterojunction under a vacuum and in the air, respectively. Different groups of treated Sb_2_Se_3_ solar cells were characterized and studied systematically, and the effects of post-annealing treatments using different scenarios on the devices are discussed.

## 2. Experimental Details

### 2.1. Preparation of Sb_2_Se_3_ Thin Film

Mo-coated glass was used as the solar cell substrates which were cleaned ultrasonically using detergent, isopropanol, ethanol and deionized water. Sb metallic precursors were then deposited onto the substrate surface via radio-frequency magnetron sputtering. Prior to deposition, the sputtering chamber was evacuated to 6.0 × 10^−4^ Pa. The sputtering power and pressure were selected as 30 W and 1 Pa, respectively. The flow rate of high-purity (>99.999%) argon (Ar) was set as 40 sccm. The substrate temperature was not specifically controlled during the sputtering process. The whole sputtering process lasted for 40 min to prepare uniform Sb precursors with a thickness of about 600 nm. After that, crystallized Sb_2_Se_3_ thin-film absorbers were grown using a post-selenization process. The as-deposited Sb metallic precursors were kept in a graphite box, which was then placed into the chamber of a vacuum tubular furnace. A pile of high-purity selenium (Se) pellets (about 0.1 g) was kept about 10 cm away from the graphite box. The selenization chamber was then heated up to 410 °C at a ramping rate of 20 °C/min. The selenization time and pressure were set as 15 min and 7 × 10^4^ Pa, respectively. The samples were finally taken out from the chamber after the furnace was naturally cooled down below 60 °C.

### 2.2. Assemble of Sb_2_Se_3_ Thin-Film Solar Cells

Cadmium sulfide (CdS) buffer layers were deposited on the as-prepared Sb_2_Se_3_ absorbers via chemical bath deposition (CBD). Aqueous solutions of CdSO_4_ (0.015 M), thiourea (0.75 M) and ammonium hydroxide (28%) were mixed with deionized water. The samples were soaked into the well-mixed solution, which was then kept in an 80 °C water bath under continuous stirring for 9 min. The thickness of the CdS buffer was around 60 nm in this work.

All the substrates were dried in an oven with a temperature of 60 °C once the CdS deposition was finished. The samples were then subjected to post-annealing treatments in vacuum and ambient air, respectively. For the vacuum annealing group, the samples were placed in a vacuum tubular furnace. The annealing temperature was increased to 325 °C in 5 min. The annealing duration was set as 5 min, and the furnace was being evacuated by a mechanical pump constantly throughout the annealing period. For the air annealing group, the samples were annealed using a hotplate. The substrates were put onto the hotplate once the temperature of it reached 325 °C. The air annealing duration was selected as 5 min as well.

**Figure 1:**
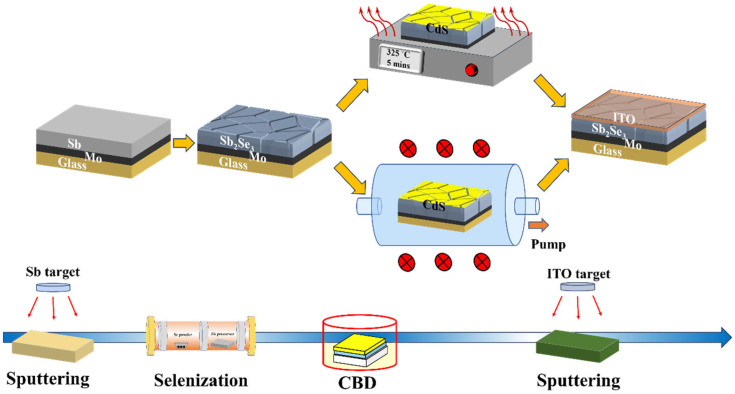
Schematic diagram of the device fabrication procedures.

After the post-annealing treatments, ITO window layers were deposited on the samples using radio-frequency magnetron sputtering. Ag electrodes were deposited onto the sample surfaces by thermal evaporation with the aid of a mask. After that, the device surface was scribed into small boxes with an identical active area (0.135 cm^2^) by knife. Ultimately, the Sb_2_Se_3_ thin-film devices with a substrate configuration of glass/Mo/Sb_2_Se_3_/CdS/ITO/Ag were assembled. The devices demonstrated excellent reproducibility as the efficiency variation between each individual sub-cell is generally less than 10%. All the devices were kept in the ambient air for over a month without any encapsulation. No apparent efficiency difference was observed for the devices before and after the storage. The flow chart of the device fabrication process is illustrated in Figure 1.

### 2.3. Characterizations

The crystal phase and morphology of the as-prepared crystallized Sb_2_Se_3_ thin films were characterized by X-ray diffraction (Ultima-iv, Rigaku, Tokyo, Japan). X-ray photoelectron spectroscopy (ESCALAB 250Xi, Thermo Scientific, Waltham, MA, USA) was utilized to measure the chemical and electronic states of the elements at the Sb_2_Se_3_/CdS heterojunction interface. All the XPS spectra were calibrated to the C1 s peak located at 284.80 eV. The current density-voltage (*J-V*) measurements of the solar cells were carried out using a Keithley 2400 multi-meter, Keithley Instruments, Cleveland, OH, USA, under the standard test conditions (AM 1.5G, 100 mW/cm^2^). The external quantum efficiency (EQE) measurements of the devices were taken by a Zolix SCS101 system and Keithley 2400 multi-meter. Capacitance-voltage (*C-V*) profiling was recorded at a frequency of 10 kHz and an AC amplitude of 30 mV, respectively in the dark. In addition, a DC bias voltage was applied from −1 V to 0.3 V for the *C-V* profiling. For the drive-level capacitance profiling (DLCP) measurements, an AC amplitude from 10 to 150 mV and a DC bias voltage from −0.25 V to 0.25 V were applied to the devices. A Lakeshore 325 temperature controller with a temperature range from 90 K to 350 K was utilized for the temperature-dependent *V_OC_* measurements. Electrochemical impedance spectroscopy (EIS) of the devices was measured using a CHI600E electrochemical workstation.

## 3. Results and Discussions

**Figure 2:**
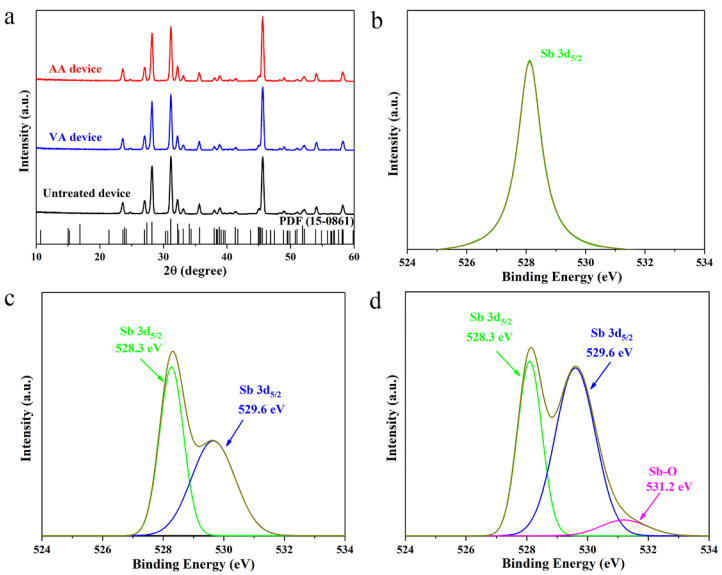
(**a**) XRD patterns of the untreated and annealed Sb_2_Se_3_ thin films. XPS spectra of Sb 3d peaks for the representative untreated (**b**), VA (**c**) and AA (**d**) Sb_2_Se_3_ thin films.

The Sb_2_Se_3_/CdS substrates that underwent annealing in vacuum and in air are denoted as VA device and AA device, respectively. XRD characterizations were conducted to figure out whether the CdS annealing treatments would influence the morphology and crystal phase of the Sb_2_Se_3_ absorber. For the VA device and AA device, CdS buffer layers were firstly etched away from the substrate surfaces using low-concentration HCl (~5%) prior to XRD and XPS measurements. The XRD patterns of the untreated and annealed samples are provided in Figure 2a. All the samples presented diffraction peaks that match perfectly with orthorhombic phases of Sb_2_Se_3_. In addition, strong (211), (221) and (002) peaks can be well observed in each sample category, indicating that quasi-vertically oriented crystal grains are dominant, which is beneficial for the transport of photogenerated carriers along the Sb_2_Se_3_ absorbers. Consistent with our previous work [17], no obvious peak shifting could be observed for the two annealed substrates, suggesting that significant change in the lattice parameters of the Sb_2_Se_3_ absorbers caused by elemental diffusion might not be possible. We then performed XPS characterizations to dissect the CdS annealing effects on chemical and electronic states of the elements at the Sb_2_Se_3_/CdS heterojunction interface. Peak fittings were carried out using a Gaussian–Lorentzian line shape to investigate the surface chemistry of the Sb_2_Se_3_ thin films. The distinct single sharp peak located at 528.2 eV in Figure 2b can be well assigned to the antimony Sb 3d in the untreated sample without any impurity or oxidized species [32]. The corresponding Sb 3d spectra of the VA and AA substrates are given in Figure 2c,d. A doublet is spotted in the Sb 3d spectrum of the VA sample, where the peaks at 528.3 eV and 529.6 eV can be ascribed to the antimony Sb 3d in the original Sb-Se bonds and newly formed Sb-S bonds on the sample surface, respectively [33]. The formation of new Sb-S bonds is due to the reaction of Sb_2_Se_3_ lattice and S^2−^ ions diffused from the CdS buffer layer. This is consistent with the finding from our previous work [34]. No oxidized species can be detected as expected since the annealing process was carried out under vacuum where few oxygen molecules were involved. However, an additional peak located at 531.2 eV is identified in the Sb 3d spectrum for the AA sample, which can almost certainly be attributed to Sb-O bonds in the Sb_2_O_3_ oxides [32].

**Figure 3:**
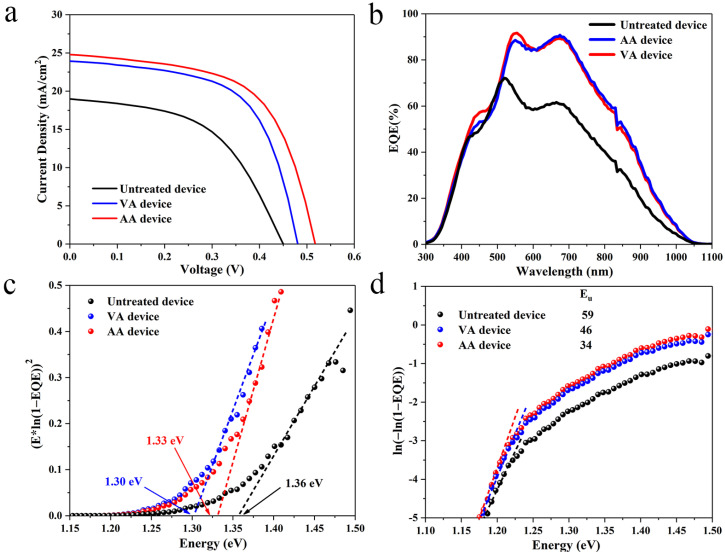
**Photovoltaic performance of devices**. (**a**) *J-V* curves of the devices, (**b**) EQE of the devices, (**c**) Bandgap derived from the EQE data of the devices, (**d**) Urbach energy derived from the EQE data of the devices.

The *J-V* curves of the devices are shown in Figure 3a. The untreated device presented a relatively low PCE of 4.4% with a *J_SC_* of 18.99 mA/cm^2^, a *V_OC_* of 0.45 V and an FF of 51.49%. The overall performances of the annealed devices were both greatly improved as the PCE of the VA device and AA device was increased to 6.92% and 7.62%, respectively. Detailed photovoltaic parameters of all the samples are summarized in Table 1. It is worth noting that the *J_SC_* and FF of the two annealed devices are close to each other whilst the *V_OC_* of the AA device is clearly larger than that of the VA device, leading to a higher PCE for the AA device. The external quantum efficiency (EQE) spectra of the devices are illustrated in Figure 3b. Obviously both the annealed devices exhibited much stronger photoresponses than the untreated device over the whole region, indicating that both the Sb_2_Se_3_ bulk film quality and the heterojunction quality were greatly improved due to the annealing treatments. The EQE curves for the two annealed devices are nearly overlapping with each other in the long wavelength region (>600 nm), whereas the photoresponse of the AA device is slightly higher than that of the VA device in the short wavelength region. This is an indication that compared to the VA device, interfacial recombination at the Sb_2_Se_3_/CdS heterojunction was moderated for the AA device [19]. The bandgaps of all the Sb_2_Se_3_ thin films are directly derived from the EQE curves (Figure 3c), and bandgap shrinkage can be found for both annealed samples. Further, the Urbach energy (*E_U_*) of the devices was estimated from the EQE data as well (Figure 3d) to evaluate the band tailing effect that usually originates from detrimental defects at the heterojunction interface [35]. A substantial decrease in *E_U_* was observed for both annealed devices where the AA device possesses the smallest *E_U_* of 34 meV among them, suggesting that the recombination losses induced by interfacial defects were effectively suppressed using the air annealing scenario.

**Figure 4:**
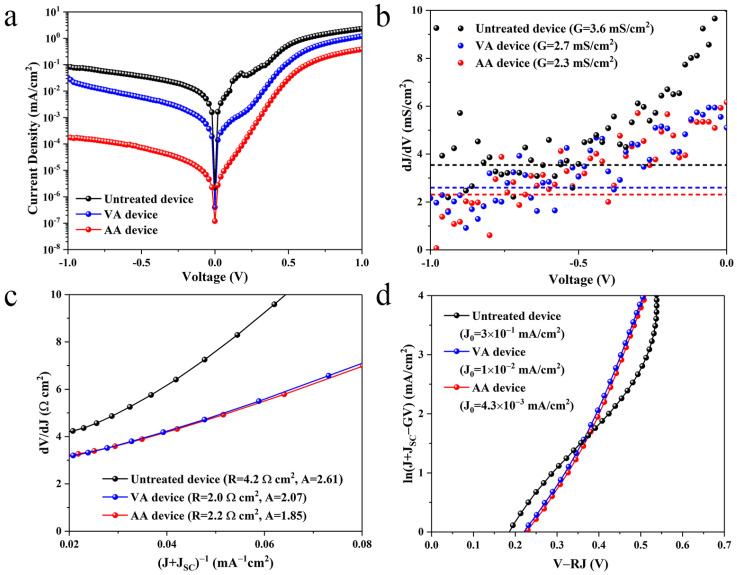
**Electrical behaviors of the representative untreated and annealed devices.** (**a**) Dark J–V curves of the devices, (**b**) conductance *G* of the devices (shown as the dotted lines), (**c**) series resistance *R_S_* and ideality factor *A* of the devices, (**d**) reverse saturation current density *J*_0_ of the devices.

Since the Sb_2_Se_3_ thin films were prepared under identical conditions, here we assume that defect states within the Sb_2_Se_3_ absorber layer are comparable for all the devices, whilst the main discrepancy in device performance came from the extent of interfacial recombination. Therefore, in order to further quantify interfacial recombination for each sample category, the diode parameters of the devices were measured in the dark, and the results are provided in Figure 4 and Table 1. Compared to the untreated device, both annealed devices exhibited a significant decrease in series resistance *R_S_*, ideality factor *A* and reverse saturated current density *J*_0_, implying positive effects of the annealing treatments on the device heterojunction quality [19]. To be more specific, lower values of *A* and *J*_0_ from the AA device suggests that deep defect-induced recombination at the interface was suppressed even more effectively than the VA device, possibly due to interfacial defect passivation by oxygen [30].

**Figure 5:**
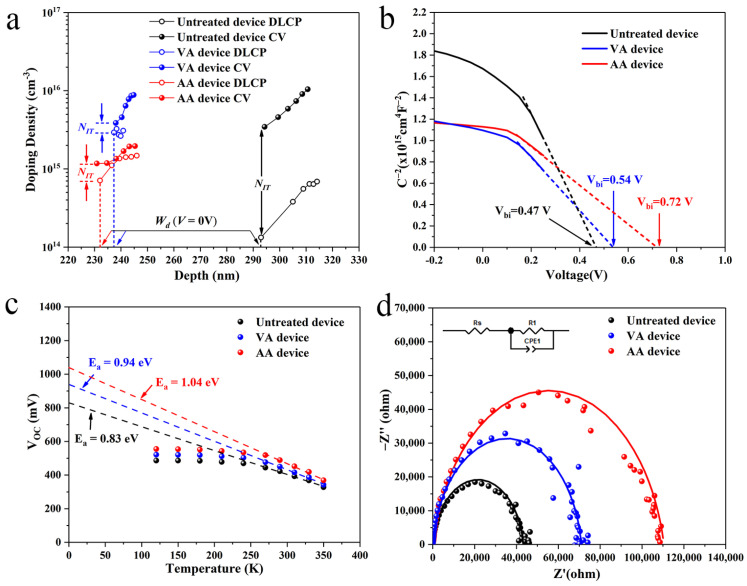
(**a**) *C-V* and *DLCP* profiling of the devices, (**b**) *V_bi_* derived from the *C-V* characterization for the devices, (**c**) Temperature-dependent VOC measurements of the devices, (**d**) Nyquist plots of the devices.

Capacitance–voltage profiling (*C-V*) and drive-level capacitance profiling (*DLCP*) are very useful tools to estimate defect density for solar cells [12,13]. The interfacial defect density of the device is often derived from the subtraction of *DLCP* data from the *C-V* data [13]. Figure 5a shows the *C-V* and *DLCP* profiling data for all the devices. Exact carrier density, as well as the calculated interfacial defect density (*N_i_*) of the devices are summarized in Table 2. The values of *N_i_* have been greatly reduced for both of the annealed devices. In addition, the AA device presents a smallest *N_i_*, which is an order of magnitude lower than that of the untreated device. Such a prominent decrease of *N_i_* for the AA device indicates oxygen might act as a defect passivator at the heterojunction interface, mitigating non-radiative recombination in this area. The built-in voltage *V_bi_* of each device was estimated by plotting *C*^−2^ against voltage *V*, as given in Figure 5b. A much larger *V_bi_* of 0.72 V is obtained for the AA device, which would naturally contribute to the enlarged *V_OC_* of the device. A temperature-dependent open-circuit voltage (*V_OC_*-*T*) measurement, which is often used as a quantitative indicator in interfacial recombination analysis, was also taken to investigate the severity of interfacial recombination of our devices (Figure 5c). The recombination activation energy (*E_a_*) for each device is obtained by extrapolating *V_OC_* to the *Y*-axis. Apparently, there is a notable discrepancy between the *E_a_* and the bandgap (estimated in Figure 3c) for each sample, indicating that the performance losses could be mainly attributed to the non-radiative recombination at the Sb_2_Se_3_/CdS interface [26]. The discrepancies between the *E_a_* and the bandgap could easily be calculated as 0.53 V, 0.36 V and 0.29 V for the untreated, VA and AA devices, respectively. The lowest value also suggests the interfacial recombination losses in the AA device were mitigated most effectively among all the samples, echoing the findings from the previous *C-V/DLCP* analyses. Finally, electrochemical impedance spectroscopy (EIS) measurements were applied to the devices to extrapolate the recombination resistance (*R_rec_*), which can be directly calculated from the diameters of the arcs in Figure 5d. The estimated *R_rec_* values for the untreated, VA and AA devices are 43,208, 70,847 and 110,020 Ω, respectively. The much enlarged *R_rec_* of the AA device suggests that non-radiative recombination originated from the heterojunction interface within the device was moderated by oxygen once an air annealing treatment was applied. Herein, it is interesting to compare our results with the ones reported by Weiss [36], where a similar post-annealing treatment in air was applied to the Sb_2_Se_3_/CdS substrate. Significant enhancement of *V_OC_* induced by interfacial recombination suppression can be observed in both studies. However, Weiss found that such a *V_OC_* improvement was accompanied by a sacrifice of *J_SC_*, leading to poor device efficiency. This apparently contradicts our condition where a prominent *J_SC_* increase was also found for the AA device. We tentatively attribute this clear contradiction to the possible difference of band alignment at the interface caused by experimental discrepancy of the two works. Heating up the Sb_2_Se_3_/CdS substrate would certainly modify the band alignment at the heterojunction and thus affect *J_SC_* of the devices. More detailed works are required to further clarify the obvious discrepancy between the two works.

## 4. Conclusions

In summary, post-annealing treatments were applied to the Sb_2_Se_3_/CdS heterojunction under a vacuum and in the air, respectively, to investigate the oxygen effect on post-annealing and thus the overall performance of the final device. It was found that the air-annealed device exhibited the highest PCE with a much-increased *V_OC_* of 0.517 V. Various analytical techniques were utilized to characterize the key photovoltaic parameters and non-radiative recombination behaviors of the devices. Data revealed that interfacial recombination was significantly suppressed for the AA device. Further, passivation of deep defects at the Sb_2_Se_3_/CdS interface was also evidenced by *C-V/DLCP* characterizations. Oxygen is believed to behave as a defect passivator at the heterojunction interface, mitigating non-radiative recombination of the AA device. Ultimately, a competitive PCE of 7.62% was achieved for the champion device.

## Figures and Tables

**Table 1:** The photovoltaic and diode parameters of the untreated and annealed devices.

Device	*V_OC_* (V)	*J_SC_* (mA/cm^2^)	FF (%)	PCE (%)	*R_S_* (Ω·cm^2^)	*A*	*J*_0_ (A/cm^2^)
Untreated device	0.450	18.99	51.49	4.40	4.2	2.61	3 × 10^−1^
VA device	0.481	23.93	60.12	6.92	2.0	2.07	1 × 10^−2^
AA device	0.517	24.80	59.44	7.62	2.2	1.85	4.3 × 10^−3^

**Table 2:** Summary of the *C-V* and *DLCP* results of the untreated and annealed devices.

Device	*N_CV_* (cm^−3^)	*N_DLCP_* (cm^−3^)	*N_i_* (cm^−3^)	*W_d_* (nm)
Untreated device	3.44 × 10^15^	1.32 × 10^14^	3.31 × 10^15^	231
VA device	3.87 × 10^15^	2.93 × 10^15^	9.40 × 10^14^	238
AA device	1.17 × 10^15^	7.10 × 10^14^	4.60 × 10^14^	294

## Data Availability

Data will be made available on request.

## References

[1] Pourasl H.H., Barenji R.V., Khojastehnezhad V.M. (2023). Solar energy status in the world: A comprehensive review. Energy Rep..

[2] Yu Y.L., Bai X.J., Li S.Y., Shi J.H., Wang L., Xi F.S., Ma W.H., Deng R. (2023). Review of silicon recovery in the photovoltaic industry. Curr. Opin. Green Sust..

[3] Ranjan S., Balaji S., Panella R.A., Ydstie B.E. (2011). Silicon solar cell production. Comput. Chem. Eng..

[4] Green M.A., Dunlop E.D., Yoshita M., Kopidakis N., Bothe K., Siefer G., Hao X. (2023). Solar cell efficiency tables (version 63). Prog. Photovolt. Res. Appl..

[5] Metaferia W., Schulte K.L., Simon J., Johnston S., Ptak A.J. (2019). Gallium arsenide solar cells grown at rates exceeding 300 µm h^−1^ by hydride vapor phase epitaxy. Nat. Commun..

[6] Radziemska E. (2003). Thermal performance of Si and GaAs based solar cells and modules: A review. Prog. Energy Combust. Sci..

[7] Chowdhury T.A., Zafar M.B., Islam M., Shahinuzzaman M., Islam M.A., Khandaker M.U. (2023). Stability of perovskite solar cells: Issues and prospects. RSC Adv..

[8] Liu N.X., Niu X.X., Chen Q., Zhou H.P. (2020). Towards commercialization: The operational stability of perovskite solar cells. Chem. Soc. Rev..

[9] Kim J.Y., Lee J.W., Jung H.S., Shin H., Park N.G. (2020). High-efficiency perovskite solar cells. Chem. Rev..

[10] Tian J.J., Xue Q.F., Yao Q., Li N., Brabec C.J., Yip H.L. (2020). Inorganic halide perovskite solar cells: Progress and challenges. Adv. Energy Mater..

[11] Zhou Y., Wang L., Chen S.Y., Qin S.K., Liu X.S., Chen J., Xue D.J., Luo M., Cao Y.Z., Cheng Y.B. (2015). Thin-film Sb_2_Se_3_ photovoltaics with oriented one-dimensional ribbons and benign grain boundaries. Nat. Photon..

[12] Tang R., Zheng Z.H., Su Z.H., Li X.J., Wei Y.D., Zhang X.H., Fu Y.Q., Luo J.T., Fan P., Liang G.X. (2019). Highly efficient and stable planar heterojunction solar cell based on sputtered and post-selenized Sb_2_Se_3_ thin film. Nano Energy.

[13] Wen X.X., Chen C., Lu S.C., Li K.H., Kondrotas R., Zhao Y., Chen W.H., Gao L., Wang C., Zhang J. (2018). Vapor transport deposition of antimony selenide thin film solar cells with 7.6% efficiency. Nat. Commun..

[14] Li Z.Q., Liang X.Y., Li G., Liu H.X., Zhang H.Y., Guo J.X., Chen J.W., Shen K., San X.Y., Yu W. (2019). 9.2%-efficient core-shell structured antimony selenide nanorod array solar cells. Nat. Commun..

[15] Li K.H., Chen C., Lu S.C., Wang C., Wang S.Y., Lu Y., Tang J. (2019). Orientation engineering in low-dimensional crystal-structural materials via seed screening. Adv. Mater..

[16] Guo C., Liang X., Liu T., Liu Y., Yang L., Lai W., Schropp R.E.I., Song D., Mai Y., Li Z. (2020). 1D/3D Alloying Induced Phase Transition in Light Absorbers for Highly Efficient Sb_2_Se_3_ Solar Cells. Sol. RRL.

[17] Tang R., Chen S., Zheng Z.H., Su Z.H., Luo J.T., Fan P., Zhang X.H., Tang J., Liang G.X. (2022). Heterojunction annealing enabling record open-circuit voltage in antimony triselenide solar cells. Adv. Mater..

[18] Cong L., Shen K., Lin D.X., Cao Y., Qiu S.D., Zheng J.Z., Bao F.X., Gao Y.Y., Zhu H.B., Li Z.Q. (2020). Back Contact Interfacial Modification in Highly-Efficient All Inorganic Planar n-i-p Sb_2_Se_3_ Solar Cells. ACS Appl. Mater. Interfaces.

[19] Luo Y.D., Tang R., Chen S., Hu J.G., Liu Y.K., Li Y.F., Liu X.S., Zheng Z.H., Su Z.H., Ma X.F. (2020). An effective combination reaction involved with sputtered and selenized Sb precursors for efficient Sb_2_Se_3_ thin film solar cells. Chem. Eng. J..

[20] Liang X.Y., Guo C.S., Liu T., Liu Y.F., Yang L., Song D.Y., Shen K., Schropp R.E.I., Li Z.Q., Mai Y.H. (2020). Crystallographic Orientation Control of 1D Sb_2_Se_3_ Nanorod Arrays for Photovoltaic Application by In Situ Back-Contact Engineering. Sol. RRL.

[21] Wang L., Li D.B., Li K.H., Chen C., Deng H.X., Gao L., Zhao Y., Jiang F., Li L.Y., Huang F. (2017). Stable 6%-efficient Sb_2_Se_3_ solar cells with a ZnO buffer layer. Nat. Energy.

[22] Liu T., Liang X.Y., Liu Y.F., Li X.L., Wang S.F., Mai Y.H., Li Z.Q. (2021). Conduction Band Energy-Level Engineering for Improving Open-Circuit Voltage in Antimony Selenide Nanorod Array Solar Cells. Adv. Sci..

[23] Zhou J., Zhang X.T., Chen H.B., Tang Z.Q., Meng D., Chi K.L., Cai Y.M., Song G.X., Cao Y., Hu Z.Y. (2020). Dual-function of CdCl_2_ treated SnO_2_ in Sb_2_Se_3_ solar cells. Appl. Surf. Sci..

[24] Zhao Y.Q., Wang S.Y., Li C.A., Che B., Chen X.L., Chen H.Y., Tang R.F., Wang X.M., Chen G.L., Wang T. (2022). Regulating deposition kinetics via a novel additive-assisted chemical bath deposition technology enables fabrication of 10.57%-efficiency Sb_2_Se_3_ solar cells. Energy Environ. Sci..

[25] Xue D.J., Shi H.J., Tang J. (2015). Recent progress in material study and photovoltaic device of Sb_2_Se_3_. Acta Phys. Sin..

[26] Yan C., Huang J.L., Sun K.W., Johnston S., Zhang Y.F., Sun H., Pu A.B., He M.R., Liu F.Y., Eder K. (2018). Cu_2_ZnSnS_4_ solar cells with over 10% power conversion efficiency enabled by heterojunction heat treatment. Nat. Energy.

[27] Su Z.H., Liang G.X., Fan P., Luo J.T., Zheng Z.H., Xie Z.G., Wang W., Chen S., Hu J.G., Wei Y.D. (2020). Device postannealing enabling over 12% efficient solution-processed Cu_2_ZnSnS_4_ solar cells with Cd^2+^ substitution. Adv. Mater..

[28] Niu X., Zhu H., Liang X., Guo Y., Li Z., Mai Y. (2017). Air-annealing of Cu(In, Ga)Se_2_/CdS and performances of CIGS solar cells. Appl. Surf. Sci..

[29] Gao S.S., Zhang Y., Ao J.P., Li X.L., Qiao S.A., Wang Y., Lin S.P., Zhang Z.J., Wang D.X., Zhou Z.Q. (2018). Insight into the role of post-annealing in air for high efficient Cu_2_ZnSn(S, Se)_4_ solar cells. Sol. Energy Mater. Sol. Cells..

[30] Liu X.S., Chen C., Wang L., Zhong J., Luo M., Chen J., Xue D.J., Li D.B., Zhou Y., Tang J. (2015). Improving the performance of Sb_2_Se_3_ thin film solar cells over 4% by controlled addition of oxygen during film deposition. Prog. Photovolt. Res. Appl..

[31] Jakomin R., Rampino S., Spaggiari G., Casappa M., Trevisi G., Canale E.D., Gombia E., Bronzoni M., Sossoe K.K., Mezzadri F. (2024). Cu-Doped Sb_2_Se_3_ Thin-Film Solar Cells Based on Hybrid Pulsed Electron Deposition/Radio Frequency Magnetron Sputtering Growth Techniques. Solar.

[32] Liu D., Tang R.F., Ma Y.Y., Jiang C.H., Lian W.T., Li G., Han W.H., Zhu C.F., Chen T. (2021). Direct hydrothermal deposition of antimony triselenide films for efficient planar heterojunction solar cells. ACS Appl. Mater. Interfaces.

[33] Lian W.T., Jiang C.H., Yin Y.W., Tang R.F., Li G., Zhang L.J., Che B., Chen T. (2021). Revealing composition and structure dependent deep-level defect in antimony trisulfide photovoltaics. Nat. Commun..

[34] Liang G.X., Luo Y.D., Chen S., Tang R., Zheng Z.H., Li X.J., Liu X.S., Liu Y.K., Li Y.F., Chen X.Y. (2020). Sputtered and selenized Sb_2_Se_3_ thin-film solar cells with open-circuit voltage exceeding 500 mV. Nano Energy.

[35] Gong Y.C., Qiu R.C., Niu C.Y., Fu J.J., Jedlicka E., Giridharagopal R., Zhu Q., Zhou Y.G., Yan W.B., Yu S.T. (2021). Ag incorporation with controlled grain growth enables 12.5% efficient kesterite solar cell with open circuit voltage reached 64.2% Shockley–Queisser limit. Adv. Funct. Mater..

[36] Weiss T.P., Bacho I.M., Zuccalà E., Melchiorre M., Valle N., Adib B.E., Yokosawa T., Spiecker E., Bachmann J., Dale P.J. (2023). Post-deposition annealing and interfacial atomic layer deposition buffer layers of Sb_2_Se_3_/CdS stacks for reduced interface recombination and increased open-circuit voltages. Prog. Photovolt. Res. Appl..

